# Deep Question Answering for protein annotation

**DOI:** 10.1093/database/bav081

**Published:** 2015-09-16

**Authors:** Julien Gobeill, Arnaud Gaudinat, Emilie Pasche, Dina Vishnyakova, Pascale Gaudet, Amos Bairoch, Patrick Ruch

**Affiliations:** ^1^BiTeM group, University of Applied Sciences-HEG, Library and Information Sciences,; ^2^SIBTex group, Swiss Institute of Bioinformatics,; ^3^University and Hospitals of Geneva, Division of Medical Information Sciences, Geneva, Switzerland and; ^4^Calipho group, Swiss Institute of Bioinformatics

## Abstract

Biomedical professionals have access to a huge amount of literature, but when they use a search engine, they often have to deal with too many documents to efficiently find the appropriate information in a reasonable time. In this perspective, question-answering (QA) engines are designed to display answers, which were automatically extracted from the retrieved documents. Standard QA engines in literature process a user question, then retrieve relevant documents and finally extract some possible answers out of these documents using various named-entity recognition processes. In our study, we try to answer complex genomics questions, which can be adequately answered only using Gene Ontology (GO) concepts. Such complex answers cannot be found using state-of-the-art dictionary- and redundancy-based QA engines. We compare the effectiveness of two dictionary-based classifiers for extracting correct GO answers from a large set of 100 retrieved abstracts per question. In the same way, we also investigate the power of GOCat, a GO supervised classifier. GOCat exploits the GOA database to propose GO concepts that were annotated by curators for similar abstracts. This approach is called deep QA, as it adds an original classification step, and exploits curated biological data to infer answers, which are not explicitly mentioned in the retrieved documents. We show that for complex answers such as protein functional descriptions, the redundancy phenomenon has a limited effect. Similarly usual dictionary-based approaches are relatively ineffective. In contrast, we demonstrate how existing curated data, beyond information extraction, can be exploited by a supervised classifier, such as GOCat, to massively improve both the quantity and the quality of the answers with a +100% improvement for both recall and precision.

**Database URL:** http://eagl.unige.ch/DeepQA4PA/

## Introduction

**Figure 1.**
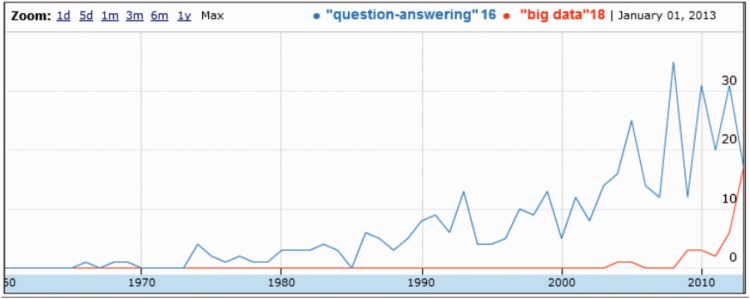
Evolution of the number of documents dealing with ‘QA’ in MEDLINE, compared with ‘Big Data’.

Biomedical professionals have access to a growing amount of literature. But when they have a precise question, they often have to deal with too many documents to efficiently find the appropriate answers in a reasonable time. Indeed, 80% of the abstracts read by Medical Literature Analysis and Retrieval System Online (MEDLINE) users appear on the first result page, which only contains the 20 most recent citations, while 50% of the searches return >20 results (1). To face this literature overload, the need for automatic assistance has been largely pointed out, and computational tools that retrieve and extract factual information are providing powerful new instruments for managing search results and staying on top of the torrent of publications (2). In particular, ontology-based search engines in MEDLINE have begun to introduce semantics in search results. These systems still display documents but users visualize sets of citations according to concepts which are extracted from their abstracts. GoPubMed (3) and EBIMed (4) are popular examples of such ontology-based search engines. Beyond this, question-answering (QA) systems are argued to be the next generation of semantic search engines (5). Figure 1 illustrates how QA has been a field of interest for several years in the biomedical literature. QA search engines do not display documents but directly concepts which were extracted from the results, and these concepts are supposed to answer the user’s question formulated in natural language. Engine for question-Answering in Genomics Litterature (EAGLi) (6), our locally developed system, is an example of such QA search engines. Thus, both ontology-based and QA search engines include the crucial task of efficiently extracting answers from the result set, i.e. a set of documents. This task is sometimes called distant reading or macro-reading, in contrast with micro-reading—or classification, or categorization, or indexing—which is a traditional Natural Language Processing task that aims at extracting concepts from a single document (7). This article focuses on macro-reading of MEDLINE abstracts, or how to extract complex genomics concepts from a set of retrieved abstracts in the framework of a QA search engine.

Several experiments have been reported to find the best way to extract ontology terms out of a single MEDLINE abstract. In particular (8), compared the performances of six classification systems for reproducing the manual Medical Subject Headings (MeSH) annotation of a MEDLINE abstract. The evaluated systems included two dictionary-based classifiers (sometimes also called thesaurus-based or morphosyntactic systems). These classifiers aim at finding ontology terms literally in the text, by alignment of words or strings, ignoring the contextual knowledge in the abstract. The Trieschnigg *et al*.’s (8) study also included a supervised classifier, which aims at exploiting a knowledge base that contains already annotated abstracts and inferring the annotation thanks to machine learning. These supervised approach thus are able to propose answers that are inferred from the knowledge base and that are not explicitly mentioned in the text. The authors concluded that, for MeSH concepts, the supervised approach outperformed the dictionary-based ones. The past BioCreative evaluation campaigns also designed tasks of Gene Ontology (GO) concepts assignment, a controlled vocabulary for the characterization of proteins functions (9). In the 2005 edition, the dictionary-based approaches clearly outperformed the supervised ones but the lack of sufficient training data was pointed out (10). Interestingly, 8 years later, the 2013 edition of the campaign proposed to revisit GO assignment tasks with different outcomes: this time, supervised systems that exploit curated data outperformed dictionary-based approaches (11). Yet, the macro-reading task we are interested in this article can be seen as fundamentally different, as it looks for the best way to extract and combine ontology terms from a set of MEDLINE abstracts.

**Figure 2.**
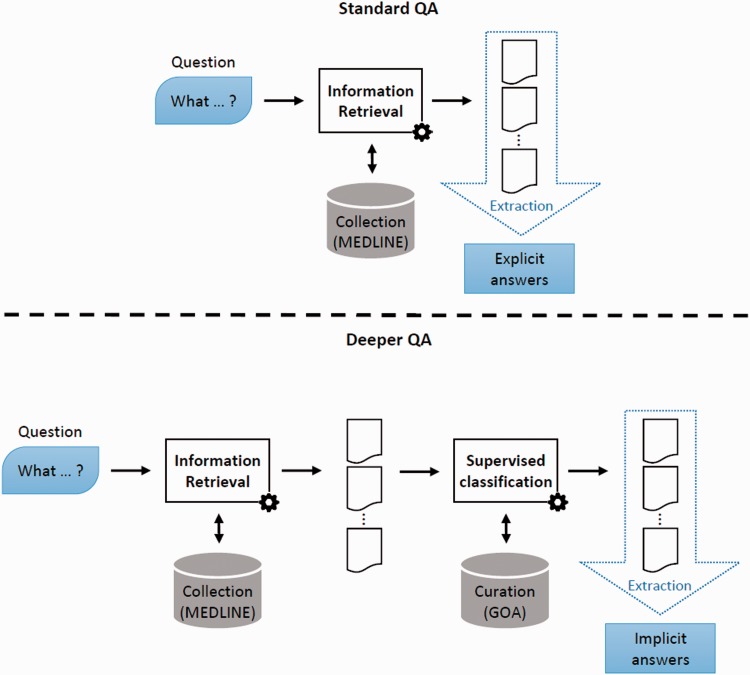
Deep QA. In standard QA, answers are extracted from some retrieved documents. In Deep QA, curated data are exploited to build a supervised classification model, which is then used to generate answers.

QA systems are commonly defined according to the types of answers they are likely to output. The main output types include factoid, list or definitional answers (5). For these different outputs, specific metrics have been developed, e.g. Top Precision for factoid answers or passage-based metrics for answers containing a full sentence (12). Several methods and combination of methods have been proposed to perform those tasks. It has then been shown that factoid QA—which commonly includes list QA—can be performed by exploiting the redundancy phenomenon (6). Such redundancy-based systems capitalize on the availability of the following resources: question understanding (13), large corpora (14) and last but not least terminological resources (5). In this article, we introduce a new type of factoid QA, deep QA, which aims at providing answers which cannot be found in any corpora. Indeed, such answers are well-defined concepts (e.g. protein functions), which can be comprehensively listed in a vocabulary, however such concepts are usually not found explicitly in documents; therefore, standard QA systems are not able to output these answers. We propose to coin a new name for those factoid—yet more complex—QA tasks: deep QA. Beyond marketing efforts, which do not always attempt to clearly define their foundational ideas (15), we propose the following definition: Deep QA is the ability of a QA engine to propose answers found in no corpus. Deep QA is needed to answer questions such as ‘What molecular functions are associated with protein X ?’. Such questions are simple regarding their structure, they are basically ‘What’ questions but traditional factoid QA systems, which are based on redundancy and dictionaries, cannot find the relevant answers. Figure 2 illustrates this difference between standard QA, for which explicit answers are found in retrieved documents, and deep QA, for which implicit answers are found in the output of a supervised classifier applied a posteriori on these retrieved documents.

### Objectives and study design

The issue addressed in this article is the extraction of GO answers from MEDLINE abstracts, in the framework of a QA system that exploit retrieved documents to output answers. As in Trieschnigg *et al*. (8) work, we first evaluate and compare a dictionary-based and a supervised classifiers for the task of assigning GO concepts to one MEDLINE abstract, and this is what we call micro-reading. Then, we evaluate and compare these classifiers for the task of assigning GO concepts to a set of 100 MEDLINE abstracts, in a QA workflow, and this is macro-reading. This two-step design is crucial as we aim at comparing the performances of the classifiers when scaling up from one citation to a set of citations. The supervised macro-reading with GOCat is what we precisely propose to call deep QA, as it adds a step in the QA workflow. For the macro-reading evaluation, we have derived from publicly available biological databases two benchmarks of 50 questions related to proteomics and biochemistry, and we used our local QA engine EAGLi. The classifiers were applied to 100 abstracts retrieved by PubMed for each question, and the most frequent GO concepts assigned with the hundred abstracts were proposed as answers.

Thus, we compare dictionary-based and supervised GO classifiers for a micro and a macro-reading task in MEDLINE. In particular, we pay attention to the role played by the redundancy of information in MEDLINE. Exploiting redundancy in large collections for QA purposes was studied among others by Lin (13), or Banko and Brill (14) in the proceedings of a the Text REtrieval Conferences (TREC) QA Track that used the Web as a resource for extracting answers. For Brill, the greater is the answer redundancy in the source, the less important are the performances of the advanced natural language processing components of the system (such as our GO classifiers that extract answers). Such components then can be seen as interchangeable black boxes. This is the idea of allowing the data, instead of the methods, do most of the work, or the idea that data is all that matters. This idea is challenged by our study.

To compare our two local classifiers to a well-known reference, we also evaluated a second dictionary-based GO classifier for answer extraction, which is the GoPubMed classifier. Thus, three classifiers were used as ‘black boxes’ of our QA system to extract GO answers from sets of 100 retrieved abstracts. Finally, we also evaluated a vectorial search engine in MEDLINE, instead of PubMed, as ‘black box’ of our QA system for retrieving abstracts.

It is important to consider our work in the light of the intrinsic complexity of the gene function curation task. Indeed, when designing the corpus of the BioCreative IV GO task, (15) observed an inter-annotator agreement (IAA) of 9.3% (strict) for evidence sentence selection and an IAA of 47% (strict) for GO term selection.

## Material and Methods

### The three GO classifiers

In this study, we evaluate three GO classifiers: two dictionary-based systems and a supervised one. The first dictionary-based classifier was ‘EAGL’: it is described comprehensively in (16). Basically, the EAGL classifier relies on two components. The first one acts like a vector-space search engine: it indexes all GO terms and synonyms as if they were documents, then it treats the input text to find the most similar GO terms. This component is expected to provide high recall. The second component is based on a lazy pattern matching component and it aims at boosting GO terms that were recognized in the input text. EAGL showed very competitive results when it was compared to other state-of-the-art dictionary-based classifiers, as during the official BioCreative I evaluation. More recently, it also showed very competitive results in the Trieschnigg *et al*.’s (8) work when compared to MetaMap. In our experiments, we thus assume that EAGL is a state-of-the-art dictionary-based classifier.

The second dictionary-based classifier, tested in our experiments, is the tool embedded in GoPubMed (17). GoPubMed is an ontology-based search engine on top of MEDLINE: it retrieves abstracts according to the user’s query, and then it extracts MeSH and GO concepts from the result set. The GOPubMed classifier is not scientifically described, but the tool is supposed to work with ‘local sequence alignment of words of the abstract and the words of GO terms’ (18). In this study, we clearly consider the GoPubMed classifier as a ‘black box interchangeable’ with EAGL. For this study, we directly asked the GoPubMed’s administrators to provide us with the GoPubMed terms extracted in sets of abstracts, and we were charged 600 euros for the processing of approximately 8000 abstracts.

The supervised classifier is GOCat (19), a locally developed classifier which is based on the *k*-nearest neighbours algorithm (20). GOCat assigns to an unseen abstract the GO terms that are the most prevalent among the *k* most similar abstracts contained in a knowledge base. The knowledge base was designed from the GOA database, which contained 85 000 manually curated abstracts when it was accessed (1 August 2012) on (21). These abstracts were indexed with Terrier, a standard Information Retrieval engine (22), and for each input text, the *k *= 100 most lexically similar abstracts in GOA were retrieved to infer the GO terms. The underlying idea is that curated abstracts contained in GOA that share the highest lexical similarity with the input text are likely to share the annotated GO concepts. GOCat is a standalone application, which is used by several anonymous users and some well-known molecular biology databases such as COMBREX (23).

### The QA platform

**Figure 3.**
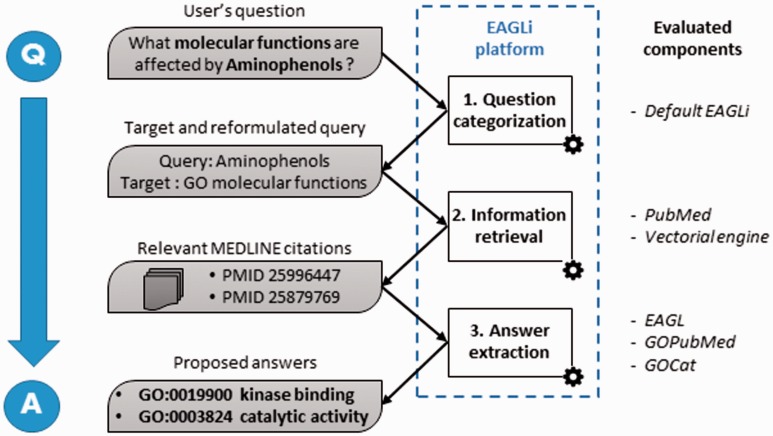
Overall workflow of the EAGLi platform. The input is a question formulated in natural language, the output is a set of candidate answers extracted from a set of retrieved MEDLINE abstracts.

For the macro-reading task, we used EAGLi, our locally developed QA system (24). This system aims at providing concepts that answer a user’s question formulated in natural language. EAGLi is composed of three independent components that are illustrated in Figure 3. First, given the user’s question, a question categorizer identifies the target set (i.e. the candidate set of possible answers) and the reformulated query which will be used by the Information Retrieval component. The target set is a subset of concepts belonging to a controlled vocabulary, which are likely to be answers to the user’s question. For instance, for the question ‘what molecular functions are affected by Aminophenols ?’, the question categorizer identifies the molecular_function axis of the GO as the target set. It means that ultimately, EAGLi will propose these and only these GO terms as answers. In our study, the target set is only of two types: (i) the molecular function axis of the GO for one of the benchmark of questions and (ii) the cellular component axis of the GO for the other benchmark. The question categorizer also outputs the reformulated query needed to retrieve the relevant documents. The reformulation is needed because non-informative words need to be discarded before querying PubMed. Thus, in the previous example, the query to PubMed only contained ‘Aminophenols’. The question categorizer was the default EAGLi component and remained unchanged during all the study. Then, given the reformulated query, the Information Retrieval component retrieves a set of relevant citations in MEDLINE. Here, we tested two search engines: first PubMed (Boolean search ordered by reverse-time) and second a vector-space (or vectorial) search engine. This vectorial search engine aims at retrieving abstracts sorted by relevance, and it was obtained by indexing a local version of MEDLINE with Terrier (22). We used Okapi BM25 as weighting scheme, which is a competitive baseline (25). In these experiments, queries were limited to the preferred name of the protein/chemical contained in the benchmarks. Finally, given the set of relevant citations and the target, the answer extraction component extracts GO terms found in the first 100 retrieved abstracts, then it computes a score for each GO term and finally outputs a list of GO candidate answers ranked by confidence scores. To assign the scores, EAGLi simply counts the number of citations containing the candidate answer. The system’s ranking principle is purely statistical: the concepts that are the most assigned to the relevant documents are the best answers.

### Experiments

Our micro-reading task consisted in assigning GO terms to a MEDLINE abstract. For evaluation purposes, we designed a so-called GOA benchmark of 1000 MEDLINE abstracts sampled from the curated section of the GOA database, and our two local GO classifiers were evaluated on their ability to extract the GO terms that were manually associated with these abstracts in the curated subset of GOA.

Our macro-reading task consisted in assigning GO terms to a set of 100 MEDLINE abstracts. For this purpose, we used EAGLi, our locally developed QA system. The three GO classifiers and the two search engines were successively embedded in the system, as illustrated in Figure 3, to perform an extrinsic evaluation. Thus, we designed two benchmarks of 50 questions by exploiting two biological databases: the comparative toxicogenomics database (CTD), which contains more than 2800 chemicals annotated with GO terms, and is available at (26) and the UniProt database, which contains millions of proteins often annotated with GO terms, and is available at (27). We randomly selected 50 entries from CTD, and linked the given chemical compounds with their annotated molecular functions, for question such as ‘what molecular functions are affected by Aminophenols ?’. These 50 questions and the expected answers are the CTD benchmark. We also randomly selected 50 entries from Uniprot, and linked the given proteins with their annotated cellular components, for questions such as ‘what cellular component is the location of ARVCF?’. These 50 questions and the expected answers are the Uniprot benchmark. The list of questions and the benchmarks used in the experiments are available in the supplementary data. The 100 questions were then submitted to EAGLi. Thus, the macro-reading evaluation was based on the classifier’s ability to extract GO terms from a set of abstracts and then propose the correct answers contained in the reference databases. Obviously, for all evaluated tasks, the classified PMIDs were discarded from the GOCat’s knowledge base.

For the micro-reading benchmark, the gold standard contained on average 2.8 annotated GO terms per abstract. For the macro-reading task, the number of possible answers contained in the gold standard was different depending on the benchmark. For the CTD benchmark, there was on average 30 relevant GO terms per question; i.e. on average 30 correct answers were expected for each question. For the UniProt benchmark, it was on average 1.3. To compute the results, we selected metrics which are designed for evaluating ranked results and which are used in routine during the TREC campaigns (28). The Top Precision (P0) is the interpolated precision at recall 0, i.e. the best precision observed at all ranks. For the QA benchmarks, we also computed Recall which is the ratio of all the possible correct answers that are returned by the QA engine. P0 tends to favour the quality of the first proposed answers. Recall tends to favour the coverage of the whole answers list. For a proteomic question that has on average 1.3 answers, we assume that the user is ready to consider the top-5 answers, thus we evaluate the Recall at Rank 5. For a chemical question that has on average 30 answers, we assume that the user is ready to see 100 answers, thus we evaluate the Recall at Rank 100. For all tasks, we considered only strict correspondence between the expected GO terms (i.e. gold file) and the evaluated answers: the systems had to output the exact GO identifiers, and no hierarchic or semantic similarities were tolerated in the evaluation (29).

The list of questions and the benchmarks used in the experiments are available in the supplementary data.

## Results and Discussion

### Micro-reading task

Table 1 presents the results obtained by our two GO classifiers (the dictionary-based categorizer EAGL and the supervised one GOCat) for the micro-reading task, i.e. the extraction of GO categories out of one single abstract. The GOA benchmark contained 1000 MEDLINE citations, manually curated in GOA. The supervised approach outperformed the dictionary-based one: +109% for P0, +117% for Recall at Rank 5. This result is not a surprise. We previously showed (19) that, thanks to the growing amount of curated data, the performances of GOCat have steadily improved across past years, while performances of EAGL is rather constant, although a massive effort was put into adding synonyms in GO.

**Table 1.** Performances for different combinations of Information Retrieval (IR) component/GO classifier for the micro-reading then the macro-reading tasks, in terms of Top Precision P0 and Recall at rank *r*

Task	Benchmark	IR component	GO classifier	P0	R at rank *r*
Micro-reading	GOA benchmark	N/A	EAGL	0.23	0.17
			GOCat	0.48* (+109%)	0.37* (+117%)
Macro-reading	CTD benchmark	PubMed	EAGL	0.34	0.15
			GOCat	0.69* (+103%)	0.33* (+120%)
			GoPubMed	0.39	0.16
		Vectorial	EAGL	0.33	0.14
			GOCat	0.66* (+100%)	0.33* (+135%)
	UniProt benchmark	PubMed	EAGL	0.33	0.45
			GOCat	0.58* (+76%)	0.73* (+62%)
			GoPubMed	0.22	0.21
		Vectorial	EAGL	0.34	0.49
			GOCat	0.58* (+70%)	0.75* (+53%)

For Recall at rank *r*, according to the average number of expected good answers for each benchmark, *r* was 5 for the GOA and the UniProt benchmarks (respectively 2.8 and 1.3 expected good answers) and 100 for the CTD benchmark (30 expected good answers). For the GOCat classifier results, improvements of performances (+ x%) are given compared with the EAGL classifier. Statistically significant improvements (*P* < 0.05) are marked up in the table with an ‘*’.

### Macro-reading task

Table 1 also presents the results obtained for the macro-reading task: extracting GO answers from a large set of 100 retrieved abstracts in the QA engine. Two components of the QA engine are studied: the answer extraction component and the Information Retrieval component. For the answer extraction component, we evaluated three GO classifiers: EAGL and GoPubMed which are dictionary-based classifiers, and the supervised one GOCat. For the Information Retrieval component, we evaluated two search engines: PubMed (Boolean search ordered by reverse-time) and our vectorial search (i.e. ordered by relevance) engine in MEDLINE.

For both benchmarks, when we focus on the results obtained with PubMed, we observe that the answers obtained with the supervised classifier (deep QA) outperformed the dictionary-based ones. When comparing the dictionary-based classifiers, we observe that GoPubMed performed better than EAGL on the CTD benchmark, while EAGL performed better than GoPubMed on the UniProt benchmark. Altogether, none of these two systems reached satisfactory results. Indeed, none of them is able to approach the performances of GOCat. From an information retrieval point of view, this result is highly significant. Indeed, several experiments using machine learning to improve information retrieval were disappointing or at least inconclusive considering effectiveness (30) and efficiency (31).

Here, the only disadvantage of GOCat is that it can only learn to detect GO terms that have already been annotated in GOA. In (19), we decomposed the GOCat performances in terms of the frequency of the GO terms. It appeared that, in GOA, the 4762 GO categories that had more than 10 assignments in GOA represented 78% of the annotations, and that most of the power of GOCat came from these terms. A minimum of 10 instances per class is often considered as a minimal number of instances to train a classifier in similar experiments (20). When applied to QA, we assume that these frequently annotated GO terms are likely to be the concepts that are useful for curators.

Interestingly, the differences between the supervised classifier GOCat and the dictionary-based classifier EAGL are linearly consistent when we scale up from the micro-reading to the macro-reading task. For micro-reading (extracting GO terms from one abstract) GOCat has a P0 of 0.48 versus 0.23 for EAGL (+109%). For macro-reading (extracting GO terms from 100 retrieved abstracts), the P0 of the QA engine is 0.69 with GOCat versus 0.34 for EAGL in the CTD benchmark (+103%) and 0.58 versus 0.33 for the Uniprot benchmark (+76%).

### Role of the search engine

Focusing on the impact of the search engine, no significant difference is observed when using PubMed or the vectorial search engine. It suggests that the choice of the search engine has no impact on the QA performances. As argued by Lin (13), when data redundancy is sufficient, the selection and parametrization of the search engine as little impact on QA. The ability to detect the answer, which relies on syntactic and semantic analysis becomes far more important that the search effectiveness. More precisely, the targeted answer type and the availability of large terminological descriptors to cover the answering space seems to play a more crucial role that the search engine and its ability to index virtually any large document collection. Beyond terminologies, curation is another form of a priori knowledge which can be exploited for answering complex questions with QA engines, as in deep QA.

### Examples

Finally, Tables 2 and 3 present examples of the QA system’s output with questions from both benchmarks: ‘what cellular component is the location of ARVCF?’ and ‘what molecular functions are affected by Nitriles ?’. In these representative examples, we can observe that the QA engine proposes more correct answers with GOCat, and that these correct answers are more specific GO terms: it means that they are deeper in the ontology. If both dictionary-based classifiers are able to recognize ‘catalytic activity’ (Level 2) in the retrieved abstracts, this answer is quite general. In contrast, GOCat proposes ‘protein homodimerization activity’ (Level 4) or ‘protein serine/threonine kinase activity’ (Level 6): it is hard to consider that any dictionary-based system could literally recognize such terms in any set of scientific articles.

**Table 2.** Output of the QA engine with different classifiers used for answer extraction

Answer extractor	#	Answers proposed by the QA engine	Correctness and GO level
GoPubMed	1	GO:0005694 chromosome	
	2	GO:0005737 cytoplasm	X (3)
	3	GO:0016020 membrane	
	4	GO:0005912 adherens junction	
	5	GO:0005886 plasma membrane	X (2)
EAGL	1	GO:0005912 adherens junction	
	2	GO:0005915 zonula adherens	
	3	GO:0005923 tight junction	
	4	GO:0005886 plasma membrane	X (2)
	5	GO:0005694 chromosome	
GOCat	1	GO:0005634 nucleus	X (5)
	2	GO:0005737 cytoplasm	X (3)
	3	GO:0005886 plasma membrane	X (2)
	4	GO:0005911 cell–cell junction	
	5	GO: 0005913 cell–cell adherens junction	

The question submitted was ‘what cellular component is the location of ARVCF?’, with PubMed used as IR component. The table shows the top five most confident answers proposed by the QA engine, and if these GO terms are present in the ARVCF record in UniProtKB. The GO level is the maximum number of nodes in the GO graph between the correct term and the root. There were three associated GO terms in the gold file, all three were returned by the QA system with GOCat.

**Table 3.** Output of the QA engine with different classifiers used for answer extraction

Answer extractor	#	Answers proposed by the QA engine	Correctness and GO level
GoPubMed	1	GO:0005488 binding	X (1)
	2	GO:0004707 MAP kinase activity	
	3	GO:0004871 signal transducer activity	X (2)
	4	GO:0003824 catalytic activity	X (1)
	5	GO:0031993 light transducer activity	
	6	GO:0060089 molecular transducer activity	X (1)
	7	GO:0047322 [hydroxymethylglutaryl-CoA reductase (NADPH)] kinase activity	
	8	GO:0050405 [acetyl-CoA carboxylase] kinase activity	
	9	GO:0033736 L-lysine 6-oxidase activity	
	10	GO:0005138 interleukin-6 receptor binding	
EAGL	1	GO:0005128 erythropoietin receptor binding	
	2	GO:0018822 nitrile hydratase activity	
	3	GO:0003824 catalytic activity	X (1)
	4	GO:0004601 peroxidase activity	X (2)
	5	GO:0004096 catalase activity	
	6	GO:0052716 hydroquinone:oxygen oxidoreductase activity	
	7	GO:0000257 nitrilase activity	
	8	GO:0033968 glutaryl-7-aminocephalosporanic-acid acylase activity	
	9	GO:0004806 triglyceride lipase activity	
	10	GO:0005344 oxygen transporter activity	
GOCat	1	GO:0005515 protein binding	X (2)
	2	GO:0042803 protein homodimerization activity	X (4)
	3	GO:0008270 zinc ion binding	
	4	GO:0000287 magnesium ion binding	
	5	GO:0003677 DNA binding	X (4)
	6	GO:0003700 sequence-specific DNA binding transcription factor activity	X (2)
	7	GO:0030170 pyridoxal phosphate binding	X (3)
	8	GO:0008144 drug binding	X (2)
	9	GO:0020037 heme binding	X (4)
	10	GO:0004674 protein serine/threonine kinase activity	X (6)

The question submitted was ‘What molecular functions are affected by Nitriles?’, with PubMed used as IR component. The table shows the top 10 most confident answers proposed by the QA engine, and if these GO terms are present in the Nitriles record in the CTD database. The GO level is the maximum number of nodes in the GO graph between the correct term and the root. There were 182 possible GO terms for this question.

## Conclusion

Our QA engine with supervised macro-reading in MEDLINE achieved a top-precision ranging from 0.58 to 0.69 to answer functional proteomics questions, and Recall values up to 0.75 at R5 for questions that expected an average of 1.3 correct answers. This performance should allow biologists and biocurators to save time when accessing the literature. Finally, the debate between redundancy-based and ontology-driven approaches applied to QA is renewed with the emerging of a new type of QA. This new and more complex QA is unlikely to be solved using traditional QA architectures based solely on redundancy and syntactic–semantic resources. As exemplified with the assignment of GO functional descriptors, we argue that deep QA is more likely to support data curation in life sciences than traditional search and QA systems, which do not exploit curated databases. With the availability of a growing corpus of curated data from various types, e.g. literature (23), patents (32) and Electronic Health Records (33), deep QA is likely to advance decision- support in a wide range of life and health sciences applications. The complete pipeline for deep Question-Answering described in this article is available at http://eagl.unige.ch/DeepQA4PA/.

## Supplementary Data

Supplementary data are available at *Database* Online.

## Funding

This work was supported by the Swiss National Fund for Scientific Research (neXtPresso Grant, SNF #153437). Funding for open access charge: Funding for open access charge: University of Applied Sciences Western Switzerland, Geneva (HEG/BiTeM/HES-SO Geneve).

*Conflict of interest*. None declared.

## Supplementary Material

Supplementary Data
