# Supplementary material for: Deep Question Answering for protein annotation
Source: Database (Oxford). 2015 Sep 16;2015:bav081. doi: 10.1093/database/bav081 (PMC4572360; doi:10.1093/database/bav081)
Supplement: Supplementary Data [file supp_2015_bav081_index.html]

Deep Question Answering for protein annotation — Supplementary Data 

# Deep Question Answering for protein annotation

## Supplementary Data

files

- Supplementary Data - zip file
